# Screening of potential cytotoxic activities of some medicinal plants of Saudi Arabia

**DOI:** 10.1016/j.sjbs.2021.10.045

**Published:** 2021-10-25

**Authors:** Merajuddin Khan, Mujeeb Khan, Syed F. Adil, Hamad Z. Alkhathlan

**Affiliations:** Department of Chemistry, College of Science, King Saud University, P.O.Box 2455, Riyadh 11451, Saudi Arabia

**Keywords:** Medicinal plants, Asteraceae, Polygonaceae, Resedaceae, HepG2

## Abstract

Phytochemicals from plant extracts belong to an important source of natural products which have demonstrated excellent cytotoxic activities. However, plants of different origins exhibit diverse chemical composition and bioactivities. Therefore, discovery of plants based new anticancer agents from different parts of the world is always challenging. In this study, methanolic extracts of different parts of 11 plants from Saudi Arabia have been tested *in vitro* for their anticancer potential on human liver cancer cell line (HepG2). Particularly, for this study, plants from Asteraceae, Resedaceae and Polygonaceae families were chosen on the basis of locally available ethnobotanical data and their medicinal properties. Among 12 tested extract samples, three samples obtained from *Artemisia monosperma* stem, *Ochradenus baccatus* aerial parts and *Pulicaria glutinosa* stem have demonstrated interesting cytotoxic activities with a cell viability of 29.3%, 28.4% and 24.2%, respectively. Whereas, four plant extracts including *Calendula arvensis* aerial parts, *Scorzonera musilii* whole plant, *A. monosperma* leaves show moderate anticancer properties bearing a cell viability ranging from 11.9 to 16.7%. The remaining extracts have shown poor cytotoxic activities. Subsequently, GC-MS analysis of methanolic extracts of four most active plants extracts such as *C. comosum*, *O. baccatus*, *P. glutinosa* and *A. monosperma* detected the presence of 41 phytomolecules. Among which 3-(4-hydroxyphenyl) propionitrile **(1)**, 8,11-octadecadiynoic acid methyl ester **(2)**, 6,7-dimethoxycoumarin **(3)** and 1-(2-hydroxyphenyl) ethenone **(4)** were found to be the lead compounds of *C. comosum*, *O. baccatus P. glutinosa* and *A. monosperma*, respectively.

## 1 Introduction

Natural products have been extensively used by mankind for various purposes from centuries (Schmidt et al., 2007). Particularly, with the advancement of scientific techniques over the last century, active components of various plants could be isolated in pure form for various medicinal purposes (Harvey, 1999). So far, a variety of phytomolecules have been significantly applied as active medicines or used as model components for the development of highly potent drugs (Yuan et al., 2016). Indeed, majority of the currently approved anticancer agents are either active phytoconstituents and/or based on various natural products (structurally optimized phytomolecules) (Newman and Cragg, 2012). The indispensable role of natural products as anticancer agents have been first recognized in the 1950s which led to the discovery of several important plant based anticancer therapeutics including vincristine, vinblastine, etoposide, teniposide and paclitaxel etc. (Lee, 1999, Majolo et al., 2019). These types of plants-based chemotherapeutics are either directly isolated or derived from lead structures (Greenwell and Rahman, 2015).

So far a variety of plants have been evaluated to investigate their anti-carcinogenic properties (Nawab et al., 2011). Active phytoconstituents present in plant extracts and/or the mixture of other secondary metabolites offer potential efficacy during chemoprevention process (Dai and Mumper, 2010). Considering these potentials, initial screening of plant extracts is often carried out extensively around the globe by several research organizations in the quest of finding potential anticancer agents from plant sources (Chanda and Nagani, 2013, Ismail et al., 2012). In this regards, a variety of secondary metabolites isolated from plant extracts including taxol, camptothecin, ursolic acid, podophyllotoxin and combretastatins etc., have demonstrated excellent anticancer activity in both *in vitro* cell cultures and *in vivo* animal models (Réthy et al., 2007). Many of these compounds are also known to exert considerable antitumor activities against different cell lines (Lajter et al., 2013). While, several pure compounds have been tested for their anticancer potential. However, increasing number of studies point towards the importance of synergistic effect of composites or mixture of compounds present in the whole plant extract (Durazzo, 2017, Liu, 2003). Therefore, screening of different plants extracts to study their anticancer potential both *in vitro* and/or *in vivo* are highly desirable (Karna et al., 2012).

The Arabian Peninsula has great botanical diversity and in this region traditional medicine practice based on foods, spices and plants have been extensively applied which is commonly known as Prophetic medicine (Maideen, 2020). However, among numerous natural products, fewer plants have been subjected to scientific evaluation for their potential anticancer properties (Ijaz et al., 2017, Sheikh et al., 2017). Particularly, in terms of biodiversity, the Kingdom of Saudi Arabia represents one of the richest regions in the Arabian Peninsula due to its vast area and diverse climatic and geographical conditions (Almehdar et al., 2012). The flora of Saudi Arabia consists of a variety of important crops and medicinal plants which are traditionally applied for medicinal purposes, such as, *Acacia arabica, Artemisia judaica, Artemisia monosperma, Lantana camara, Azadirachta indica, Pulicaria glutinosa, Calligonum comosum* etc (Ullah et al., 2020).

Indeed, several ethnobotanical surveys in Saudi Arabia have revealed the dependence of a large proportion of Saudi citizens on traditional medicine whether alone or in association with modern medicine (Ali et al., 2017, Shahat et al., 2014). Although, considerable ethno-botanical data is available regarding the medicinal properties of plant species from Saudi Arabia, however, *in vitro* screening of several important medicinal plants from this region has not been performed so far. Particularly, a variety of plant extracts with potential antioxidant and other medicinal properties from Saudi Arabia such as *A. monosperma, Ochradenus baccatus, P. glutinosa, A. sieberi, Calendula arvensis* etc., which are representative plant species of this region have been rarely tested for their anticancer potential.

For instance, *A. monosperma* is a commonly existing desert plant, which is widely used in folk medicine for the treatment of hypertension, muscle spasms and parasitic worms in Saudi Arabia (Amin et al., 2019). *A. monosperma* is a perennial bushy shrub which grows up to 1-meter height containing lengthy leaves with scattered hairs and has green bud like flowers. Essential oils and extracts of this plant have been reported to exhibits various biological activities such as antimicrobial, antimalarial, antioxidant, insecticidal activities however, perusal of literature revealed that methanolic extract of this plant from Saudi Arabia has not been screened for anticancer properties (Guetat et al., 2017). Similarly, *O. baccatus* (Taily Weed) is a perennial shrub which is largely found in the southwest and focal areas of Saudi Arabia. This plant is popular in the Arabian Peninsula as a crucial source of food for animals in the desert. Due to its rich contents of antioxidant and anti-inflammatory constituents, it is also extensively used as traditional medicine for several ailments (Al-Omar et al., 2020). Indeed, the aqueous extract of *O. baccatus* from Judean Desert, Israel, has demonstrated antitumor effects against human liver cancer cells in an *in vitro* tumor model study (Thoppil et al., 2013). However, other fractions of this plant from Saudi Arabia has not been studied for potential antitumor activities. Apart from this, methanolic extract of *P. glutinosa* from Saudi Arabia has also not been evaluated for its anticancer potential, although, several species of *Pulicaria* are known to possess anticancer properties (Emam et al., 2019). Moreover, other plants such as *A. sieberi*, *Anthemis deserti, C. comosum*, *C. arvensis, Emex spinosa, Launaea capitata*, *Rumex dentatus* and *Scorzonera musilii* which were selected in this study, either not studied or have been poorly investigated for their anticancer activity.

Therefore, considering our interest in the investigation of Saudi medicinal plants, we planned this comprehensive anticancer screening study on the Saudi medicinal plant. In this study, 11 different plants species representative of Saud Arabia were chosen on the basis of locally available data on their medicinal properties and their systematic *in vitro* cytotoxic screening are being reported here against human liver cancer cell line (HepG2). Moreover, in order to investigate the type of phytomolecules which might be responsible for anticancer properties of plant extracts, we have also carried out chemical characterization of the most active plant extracts such as *C. comosum* stem, *O. baccatus* aerial parts, *P. glutinosa* stem and *A. monosperma* stem using gas chromatography-mass spectrometry (GC-MS) analysis.

## 2 Materials and methods

### 2.1 Collection and identification of plants

Various parts of 11 plants namely *A. monosperma* Del.*, P. glutinosa* Jaub. & Spach*, A. sieberi* Besser*, C. arvensis* L.*, L. capitata* (Spreng.) Dandy, *A. deserti* Boiss.*, S. musilii* Vel.*, E. spinosa* (L.) Campd., *R. dentatus* L.*, C. comosum* L.'Her. and *O. baccatus* Del. belonging to different families including Asteraceae, Polygonaceae and Resedaceae were collected from different locations of Central region of Saudi Arabia in the month of February-March-2015. Identification of plant materials was done by a plant taxonomist of herbarium division, Science college, King Saud University (KSU), Riyadh and voucher specimens of each plant were retained in herbarium division, college of science, KSU.

### 2.2 Extraction of plant materials

**Fig. 1:**
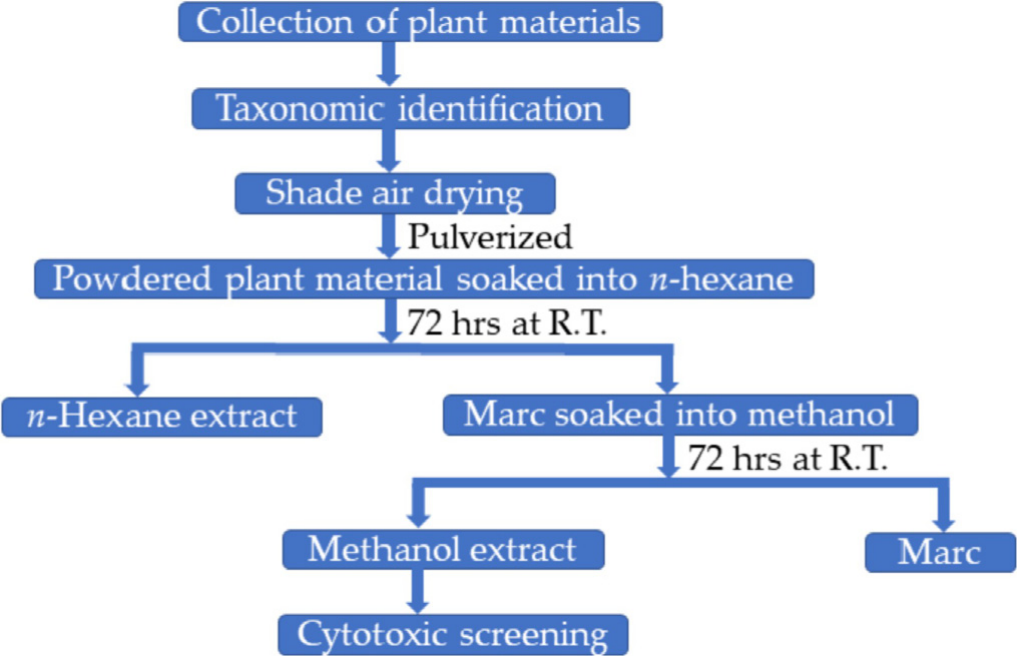
Flow chart for the extraction of plant materials for cytotoxic screening.

Collected parts of each plants were initially dried in shade, grounded and then subjected for extraction as shown in Fig. 1. The dried and pulverized plant materials were then first defatted at room temperature with hexane three times for 72 h each followed by extraction with methanol three times for 72 h each. The obtained hexane and methanolic extracts were combined and dried separately on a rotary evaporator at 45 °C under reduced pressure until solvents were completely evaporated. The resultant hexane and methanolic extracts of each plant were kept at 4 °C in dark until they were used. The methanolic extracts were subjected for the screening of anticancer activity.

### 2.3 Cytotoxic screening of plant extracts

The cytotoxicity of the twelve methanolic extracts of eleven plants were tested against HepG2 cells. The cell line was obtained from American Type Culture Collection (Manassas, VA, USA). The cells were grown in Dulbecco's modified eagle's medium (DMEM) supplemented with 10% FBS and 100 U ml^−1^ of penicillin–streptomycin. Cells were grown in a humidified environment with 5% CO_2_ at 37 °C in a CO_2_ incubator. Cells were harvested with 0.25% trypsin for sub-culturing and for further experiments, whenever needed. To test the cytotoxicity cells were seeded in a 96 well plate with 1 × 10^3^ cells per well and the plates were incubated for 24 h to allow the attachment of the cells on the surface of the wells before exposure to the test compounds. Extracts of plants were suspended in DMEM medium to give a final concentration of 50 μg mL^−1^. While, buffer was taken as control. The selection of the 50 μg mL^−1^ dosage range was based on a preliminary dose–response study (data not shown).

Following 24 h of exposure to the extracts, MTT assay was performed to check the percentage of viable cells following standard method described by Mossman (Mosmann, 1983). The assay evaluates the ability of mitochondria to reduce MTT into a blue formazan product as a measure of viability. Briefly, the MTT solution was added in an amount equal to 10% of culture volume and the plate was incubated for 3 h at 37 °C to allow the formation of a formazan from MTT. Acidified isopropanol was used to dissolve the formazan and an aliquot of 100 μL supernatant was transferred to a new 96-well plate. Finally, the absorbance was recorded at 570 nm using a microplate reader (Synergy-HT; BioTek, Winooski, VT). Percentage viability was determined by comparing the values obtained for the control with the values obtained for various treatments.

### 2.4 Gas Chromatography (GC) and Gas Chromatography–Mass Spectrometry (GC-MS) analysis of plant extracts

GC and GC-MS analysis of plant extracts were performed on an Agilent GC-MS instrument using the same system and method with slight modification as described earlier (Khan et al., 2020) and is given in detail in supplementary information.

### 2.5 Identification of phytomolecules from methanolic extracts of four most active plant extracts

The identification of different components of most active methanolic extracts of four plants were done using their GC chromatogram, retention time, elution order, peak area and by matching the mass spectra with the library entries of mass spectra databases in NIST and Wiley libraries (WILEY 9th edition, NIST-08 MS library version 2.0f).

## 3 Results and discussion

For this study, 12 methanolic extracts from different parts of 11 plant species, mostly collected from Central region of Saudi Arabia, were subjected to *in vitro* studies for their anticancer properties against liver cancer cell line (HepG2 cell line). The MTT assay shows that the methanol extracts obtained from different plants show varied degree of cytotoxicity against the HepG2 cells (Table 1). The decrease in the viability was in the range of 5.9–29.3%.

**Table 1:** Percent decrease in cell-viability of HepG2 cells following the treatment with 50 µg/ml of methanol extracts from different Saudi plants.

S. No.	Plant	Plant parts	Family	Percent decrease in Cell viability
1	*A. monosperma*	Stem	Asteraceae	29.3
2	*A. monosperma*	Leaves	Asteraceae	11.9
3	*A. sieberi*	Aerial parts	Asteraceae	7.1
4	*A. desrtii*	Whole plant	Asteraceae	6.8
5	*C. comosum*	Stem	Polygonaceae	15.9
6	*C. arvensis*	Whole plant	Asteraceae	16.7
7	*E. spinosa*	Whole plant	Polygonaceae	6.8
8	*L. capitata*	Whole plant	Asteraceae	6.1
9	*O. baccatus*	Aerial parts	Resedaceae	28.4
10	*P. glutinosa*	Stem	Asteraceae	24.2
11	*R. dentatus*	Whole plant	Polygonaceae	5.9
12	*S. musilii*	Whole plant	Asteraceae	13.9

**Fig. 2:**
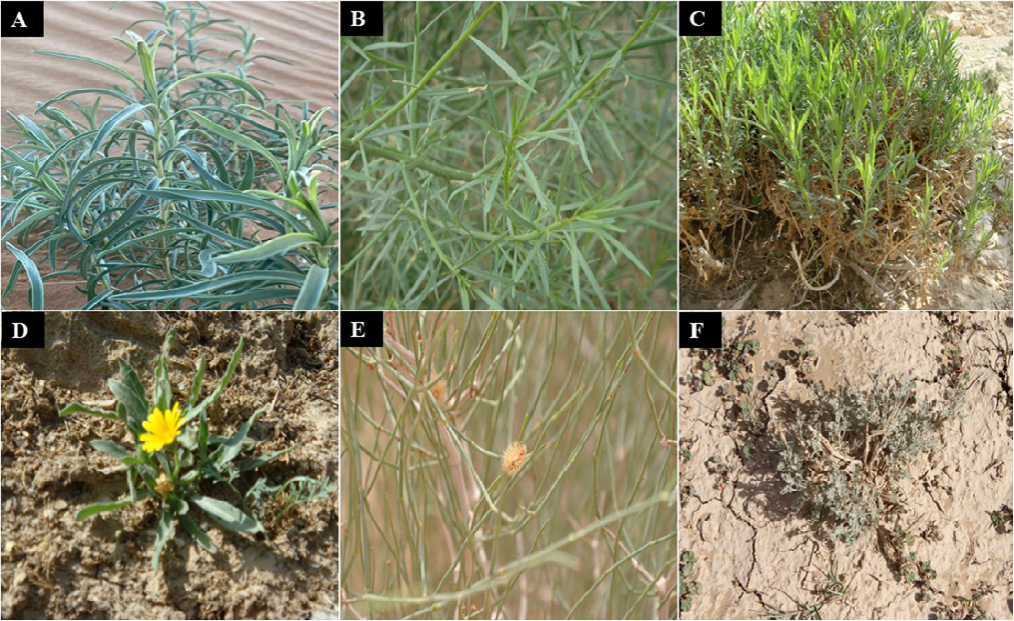
Digital images of some important Saudi plants studied for their anticancer activity: (A) *A. monosperma,* (B) *O. baccatus,* (C) *P. glutinosa,* (D) *C. arvensis,* (E) *C. comosum*, (F) *A. sieberi.*

The results in the Table 1 demonstrate that out of 12 extracts, three samples have shown a decrease of more than 24% cell viability at a concentration of 50 µg on HepG2 cells. Whereas, 9 samples have exhibited a decrease of less than 20% at a concentration of 50 µg on HepG2 cells. Among all the tested samples, the methanolic extracts of *A. monosperma* stem*, O. baccatus* aerial parts and *P. glutinosa* stem (Fig. 2) have shown highest anticancer properties with a decrease in cell viability of 29.3, 28.4 and 24.2%, respectively. On the other hand, the lowest activities were observed in the case of methanolic extracts of *R. dentatus, L. capitata*, *E. spinosa* and *A. deserti* with a decrease in cell viability ranging from 5.0 to 7.0 %.

**Fig. 3:**
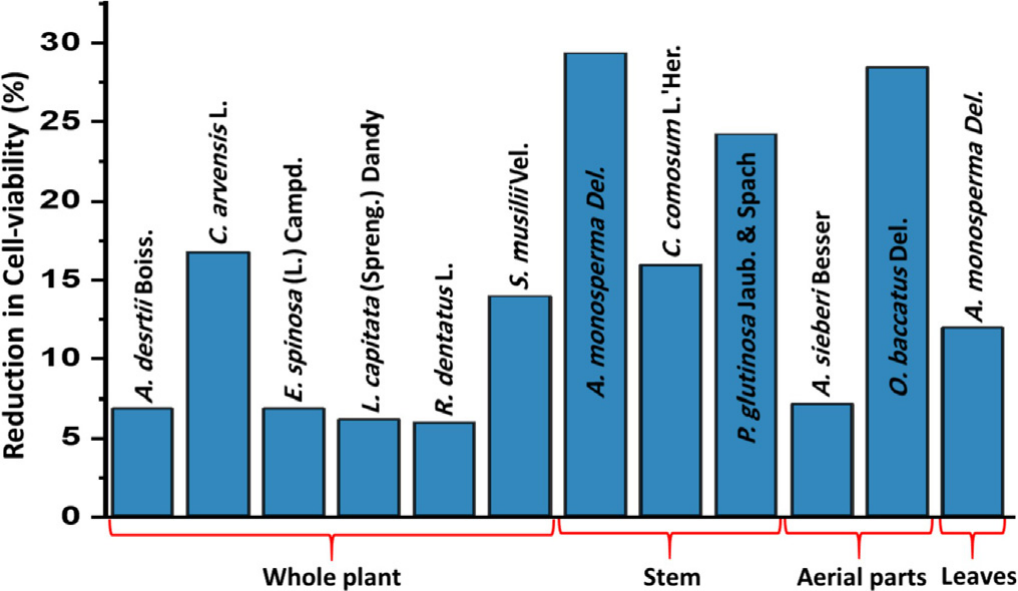
Comparative graphical illustration of reduction in cell viability of the tested extracts segregated based on plant parts.

Among various extracts, the methanolic extract of *A. monosperma* stem has exhibited highest anticancer activity with highest decrease in cell viability of ∼29.3%. While, a comparable decrease in cell viability was observed with the methanol extract of the leaves from the same plant. Another species of the same genus *A. sieberi* shows only 7.1% decrease in the cell viability (Fig. 3). *A. monosperma* is an important medicinal plant of the genus *Artemisia*. This genus is known for their essential oils which are commonly applied in cosmetics and pharmaceutical industries and also used in folk and modern medicine. Plants from this genus also possessed various biological activities including cytotoxic and antioxidant activity. Major classes of phytoconstituents found in genus *Artemisia* include terpenoids, flavonoids, coumarins, caffeoylquinic acids, sterols, and acetylenes (Bora and Sharma, 2011). In our recent study of the essential oil from the stem and leaf of *A. monosperma* we have identified 130 components. Some of the major components in the stem oil were β-pinene (36.7%), α-terpinolene (6.4%), limonene (4.8%), β-maaliene (3.7%), shyobunone (3.2%) and α-pinene (3.1%) (Khan et al., 2012).

Some studies on *A. monosperma* collected from different parts of world have identified the anticancer potential of this plant. In one study, eudesmane sesquiterpene isolated from *A. monosperma* is linked to the apoptotic cell death in the human melanoma A375 cell line (Formisano et al., 2012). While various other components isolated from this plant have shown specific *in-vitro* anticancer properties against colorectal and breast cancer cell lines (Stavri et al., 2005). Capillin (1-phenyl-2,4-pentadiyne), another polyacetylene found in *A. monosperma*, has been shown to induce apoptosis in several human tumor cell lines including colon HT29, pancreatic MIA PaCa-2, epidermoid carcinoma of the larynx HEp-2, and lung carcinoma A549 cells (Whelan and Ryan, 2004). Recently, the anticancer activity of whole plant extract of *A. monosperma* has been demonstrated against ten different human cancer cells through apoptosis (Solowey et al., 2014).

Another sample in Table 1 which displayed significant anticancer activity is methanolic extract of *O. baccatus* aerial parts which exhibited 28.4% decrease in cell viability. *O. baccatus* is an important medicinal plants of dessert region of Middle East and Africa, especially in Saudi Arabia it is abundantly available and is used in traditional medicine as an antibacterial, anti-inflammatory agent and for the treatment of sexual disorder and malaria caused by *Plasmodium falciparum* (Al-Omar et al., 2020). In previous studies ethanol extract of *O. baccatus* have shown antimicrobial activity whereas water extract of this plant has exhibited nematicidal and anticancer activities (Bhatia et al., 2015, Oka et al., 2014, Thoppil et al., 2013). Only few limited chemical investigations of *O. baccatus* have been reported. For example, different flavonoids such as quercetin, kaempferol and their various glycosides have been isolated (Barakat et al., 1991). To the best of our knowledge anticancer activity of methanolic extract of *O. baccatus* has not been reported so far. Therefore, a detailed analysis of phytochemical in methanolic extract of *O. baccatus* could guide to isolation of some lead anti-cancer phytomolecules from this abundantly available plant of Saudi Arabia.

Next sample (Table 1) is methanolic extract of *P. glutinosa* stem (PGS) which has demonstrated important anticancer property with a decrease in cell viability of ∼25%. *Pulicaria* is a relatively large genus of plants belonging to the tribe Inuleae of the daisy family Asteraceae. It comprises about 100 species distributed from Europe to North Africa and Asia, particularly around the Mediterranean. Till date, a variety of biological properties of different species of *Pulicaria* have been reported including anticancer properties of *P. crispa* and *P. orientalis* (Barnawi and Ali, 2019, Fawzy et al., 2013).

In addition to that, eupatolitin isolated from *P. undulata* has also demonstrated cytotoxic effect against MCF-7 and HepG2 cells (Hussien et al., 2016). However, to the best of our knowledge the anticancer property of *P. glutinosa* has not been reported so far. Therefore, further investigation on the cytotoxic activity of different extracts of other parts of *P. glutinosa* could potentially reveal crucial information.

Methanolic extract of *C. arvensis* aerial parts, *A. monosperma* leaves and whole plants of *S. musilii*, and *C. comosum* have demonstrated moderate anticancer activities with decrease in cell viability of 16.7%, 11.9%, 13.9% and 15.9%, respectively. Moreover, *C. arvensis* which has been traditionally used as folk medicine for treating various diseases, its extracts have also been extensively studied. For example, different parts of *C. arvensis*, such as flowers, stems and leaves extracted in a variety of solvents (hexane, chloroform, ethyl acetate, and methanol) have displayed cytotoxic activities (Abutaha et al., 2019). Among different samples, only the ethyl acetate extract of *C. arvensis* flowers demonstrated cytotoxic activity against MCF-7 and MDA-MB-231 breast cancer cells. Notably, cytotoxic activity of the methanol extract of whole plant of *C. arvensis* in this study points toward the cytotoxic potential of *C. arvensis* plant which needs to be further investigated. On the other hand, *C. comosum* is green woody perennial shrub which can be found in temperate regions of Middle East, Southern Europe, Western Asia, and North Africa (Taia and El-Etaby, 2006). This plant is medicinally used for gastric and skin diseases in traditional treatment. Different extracts such as ethanolic, methanolic and acetone extracts of *C. comosum* have been reported to have antibacterial activities (Riadh et al., 2011). Besides that, various biological activities such as anti-oxidant, anti-inflammatory, anti-ulcer, and anti-cancer activities (Alehaideb et al., 2020, Gasmi et al., 2019, Kiani et al., 2019, Soliman et al., 2020) of this plant have also been reported. Different class of phytomolecules specially flavonoids, polyphenols, terpenoids and saponins have been detected in different parts of *C. comosum* (Cheruth et al., 2016, Gasmi et al., 2019). Detail literature search on this plant suggest that not much work on the isolation of active phytomolecules has been carried out. Therefore, noticing significant anticancer activity in the present study and various pharmacological properties of this plant in previous reports make this plant a very prominent candidate for the isolation of active compounds from *C. comosum*.

In the case of *S. musilii* (with 13.9% decrease in cell viability) no single report has been found regarding the cytotoxic activity of this plant, and thus, could be potential candidate for further research. On the other hand, oil extracts of *A. monosperma* is known to act as a modulator tool for improving health status and alleviating the high risk of colon cancer complications (Sadek et al., 2015). Still, the anticancer potential of this plant has not been properly investigated, particularly plants from this species that are native to Saudi Arabia. Therefore, this plant could also be considered for further investigations. The genus *Artemisia* is one of the largest and most widely distributed genera of the family Asteracea. It is a heterogenous genus, consisting over 500 diverse species distributed mainly in the temperate zones (Bora and Sharma, 2011).

Remaining other plants in Table 1, such as *A. sieberi* aerial parts (7.1%), *A. deserti* (6.8%), *L. capitata* whole plant (6.1%), from Asteracea family and *E. spinosa* whole plant (6.9%) and *R. dentatus* whole plant (5.9%) from Polygonaceae have demonstrated poor anticancer properties with less than 10% decrease in cell viability. Notably, cytotoxic potential of extracts of some of these plants extracted in various solvents is known and well documented, for instance, ethanolic extract of *A. sieberi*, different fractions of *E. spinosa* extracts and methanol and chloroform extracts of *R. dentatus* (Abdolmaleki et al., 2015, Batool et al., 2017, Choucry, 2017, Eser et al., 2017, Soliman et al., 2014). However, other plants like *A. deserti* and *L. capitata*, have not been investigated so far to study their anticancer properties. To the best of our knowledge, no report regarding their cytotoxic activity have been found in the literature.

In order to investigate the type of phytomolecules which might be responsible for the anticancer properties of plant extracts, we have carried out chemical characterization of most active plant extracts such as *C. comosum* stem, *O. baccatus* aerial parts, *P. glutinosa* stem and *A. monosperma* stem. For this purpose, we performed gas chromatography-mass spectrometry (GC-MS) analysis of methanolic extracts of these plants. GC chromatograms obtained from methanolic extracts of each plant are given in Fig. 6 and Fig. 7, whereas their identified compounds are given in Table 2.

**Fig. 4:**
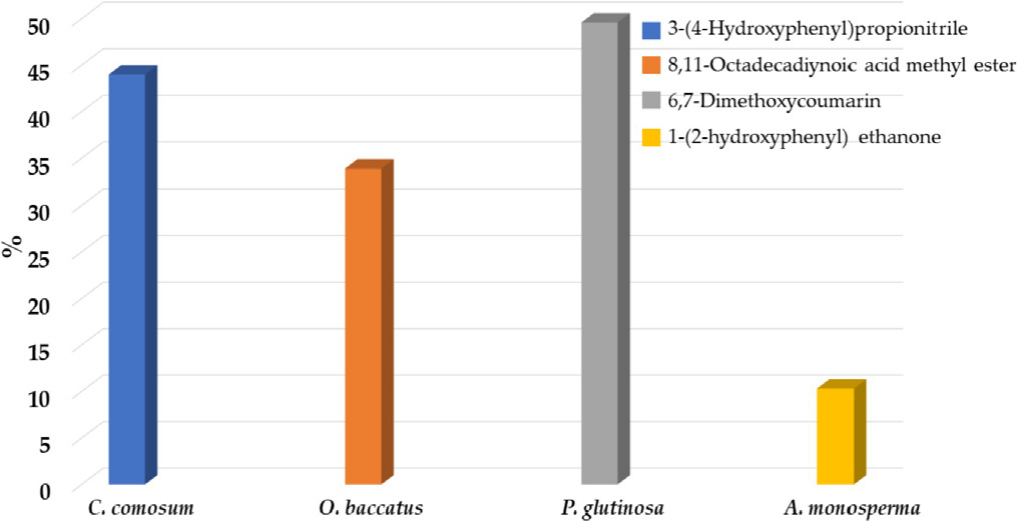
Lead compounds identified from the most active plant extracts.

**Fig. 5:**
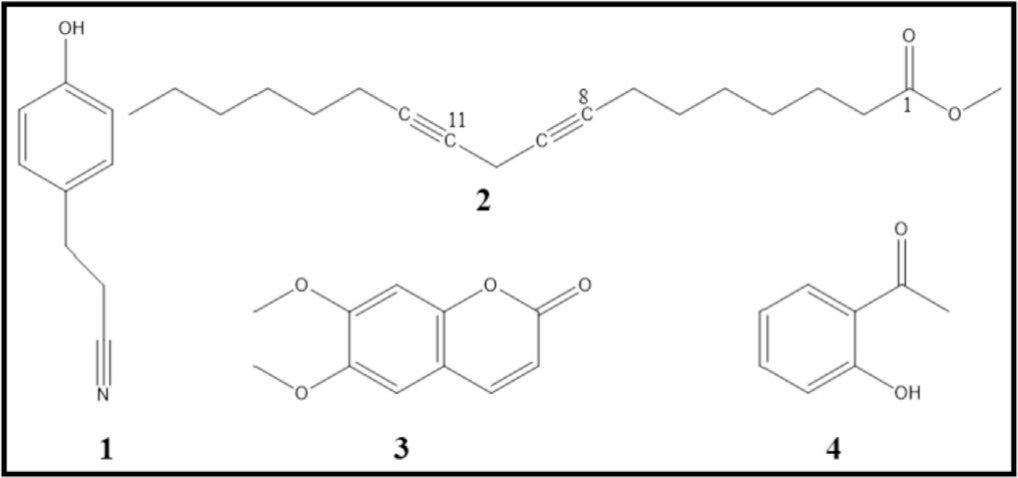
Chemical structures of lead compounds from methanolic extracts of *C. comosum* stem, *O. baccatus* aerial parts *P. glutinosa* stem and *A. monosperma* stem.

**Fig. 6:**
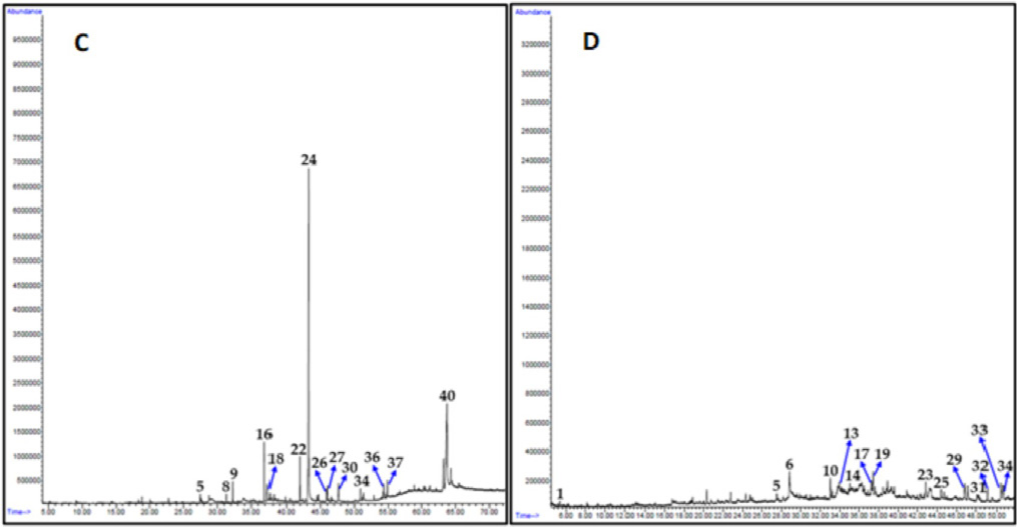
GC chromatogram of methanolic extracts of A) *C. comosum* stem, B) *O. baccatus* aerial parts. Compounds are numbered according to their position in Table 1.

**Fig. 7:**
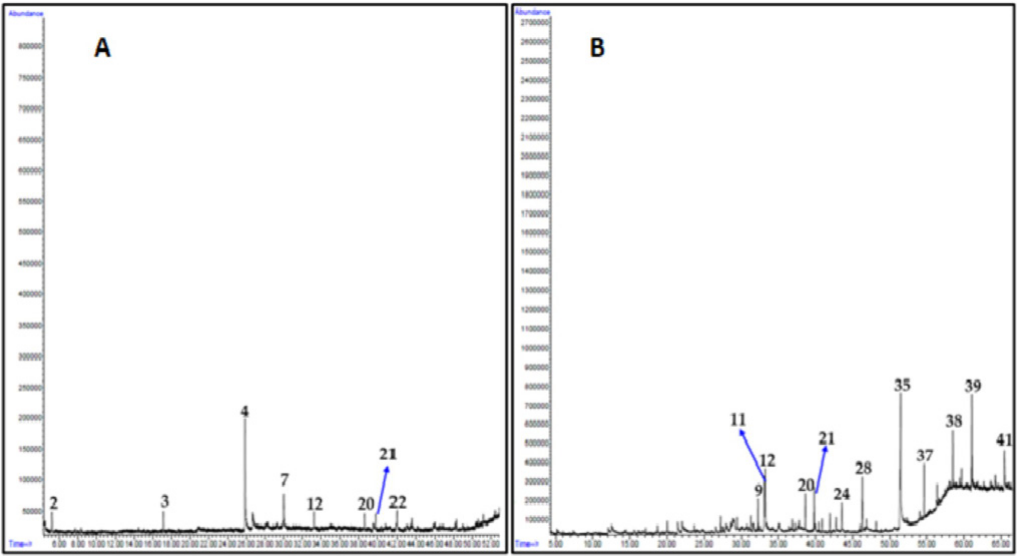
GC chromatogram of methanolic extracts of C) *P. glutinosa* stem, D) *A. monosperma* stem. Compounds are numbered according to their position in Table 1.

**Table 2:** Identified chemical compounds from the methanolic extracts of four most active plants.

S. No.	Compounds	RT (min.)	MF	MW	Peak Area (%)			
					C.C.	O.B.	P.G.	A.M.
1	2,2-Dimethoxybutane	5.18	C_6_H_14_O_2_	118	–	–	–	0.1
2	2-Methyl-1-butene oxide	5.22	C_5_H_10_O	86	6.5	–	–	–
3	6-Methyloctadecane	17.12	C_19_H_40_	268	7.4	–	–	–
4	3-(4-Hydroxyphenyl)propionitrile	25.86	C_9_H_9_NO	147	44.0	–	–	–
5	3-Methoxythiophenol	27.45	C_7_H_8_OS	140	–	–	1.1	0.5
6	1-(2-Hydroxyphenyl) ethanone	28.79	C_10_H_16_	136	–	–	–	10.3
7	6-Pentyl-5,6-dihydro-2H-pyran-2-one	30.00	C_10_H_16_O_2_	168	13.6	–	–	–
8	6-Allyl-4-methoxy-1,3-benzodioxole	31.30	C_11_H_12_O_3_	192	–	–	0.8	–
9	5-Allyl-1,2,3-trimethoxybenzene	32.25	C_12_H_16_O_3_	208	–	3.5	1.6	–
10	Tricyclo[5.2.2.0(1,6)]undecan-3-ol, 2-methylene-6,8,8-trimethyl-	33.03	C_15_H_24_O	220	–	–	–	6.7
11	4-(2,6,6-Trimethyl-1-cyclohexen-1-yl)-3-buten-2-ol	33.11	C_13_H_22_O	194	–	7.5	–	–
12	(*E*)-7-Octadecene	33.25	C_18_H_36_	252	5.9	7.6	–	–
13	9-Octadecen-12-ynoic acid, methyl ester	33.71	C_19_H_32_O_2_	292	–	–	–	5.3
14	3H-Cyclodeca[b]furan-2-one, 4,9-dihydroxy-6-methyl-3,10-dimethylene-3a,4,7,8,9,10,11,11a-octahydro-	35.02	C_15_H_20_O_4_	264	–	–	–	2.9
15	1-Heptatriacotanol	36.47	C_37_H_76_O	536	–	–	–	1.7
16	Methyl tetradecanoate	36.90	C_15_H_30_O_2_	242	–	–	4.2	–
17	1-(2,3-Dihydroxy-2-isopropenyl-2,3-dihydro-1-benzofuran-5-yl)ethanone	37.26	C_13_H_14_O_4_	234	–	–	–	6.3
18	4-((1*E*)-3-Hydroxy-1-propenyl)-2-methoxyphenol	37.30	C_10_H_12_O_3_	180	–	–	1.9	–
19	2-[4-methyl-6-(2,6,6-trimethylcyclohex-1-enyl)*hexa*-1,3,5-trienyl]cyclohex-1-en-1-carboxaldehyde	37.42	C_23_H_32_O	324	–	–	–	9.1
20	(*E*)-5-Octadecene	38.64	C_18_H_36_	252	6.5	3.9	–	–
21	(*Z*)-4-Octadecen-1-ol acetate	39.83	C_20_H_38_O_2_	310	6.7	4.8	–	–
22	*n*-Hexadecanoic acid methyl ester	41.98	C_17_H_34_O_2_	270	7.5	–	3.3	–
23	2-Allyl-1,4-dimethoxy-3-methyl-benzene	42.82	C_12_H_16_O_2_	192	–	–	–	4.6
24	6,7-Dimethoxycoumarin	43.28	C_11_H_10_O_4_	206	–	3.0	49.6	–
25	2-[4-methyl-6-(2,6,6-trimethylcyclohex-1-enyl)*hexa*-1,3,5-trienyl]cyclohex-1-en-1-carboxaldehyde	44.42	C_23_H_32_O	324	–	–	–	5.3
26	(9*Z*,12*Z*)-Octadecadienoic acid methyl ester	45.91	C_19_H_34_O_2_	294	–	–	0.9	–
27	(*Z*)-9-Octadecenoic acid methyl ester	46.05	C_19_H_36_O_2_	296	–	–	1.3	–
28	3,7,11,15-Tetramethyl-2-hexadecen-1-ol	46.31	C_20_H_40_O	296	–	6.9	–	–
29	Methyl 2-[(1*E*,3*E*)-7-hydroxy-3-methyl-1,3-octadienyl]-1,3-dimethyl-4-oxo-2-cyclohexene-1-carboxylate	47.14	C_19_H_28_O_4_	320	–	–	–	6.7
30	17-Octadecen-14-ynoic acid, methyl	47.68	C_19_H_32_O_2_	292	–	–	1.9	–
31	10-Methoxycoryn-18-en-17-yl acetate	48.10	C_22_H_28_N_2_O_3_	368	–	–	–	5.4
32	2-Thiazolamine, 4-(3,4-dimethoxyphenyl)-5-methyl-	49.04	C_12_H_14_N_2_O_2_S	250	–	–	–	5.4
33	Methyl 3-methylene-1,2,3,3a,4,4a,4b,5,6,10b-decahydrocyclopropa[3,4]cyclohepta[1,2-a]naphthalen-8-yl ether	50.44	C_18_H_22_O	254	–	–	–	8.2
34	2-Octadecyloxyethanol	50.70	C_20_H_42_O_2_	314	–	–	1.2	7.5
35	8,11-Octadecadiynoic acid methyl ester	51.47	C_19_H_30_O_2_	290	–	33.9	–	–
36	Methyl docosanoate	54.36	C_23_H_46_O_2_	354	–	–	1.0	–
37	10-Undecenoic acid, octyl ester	54.64	C_19_H_36_O_2_	296	–	5.2	1.6	–
38	1,3-Dioctadecyloxypropane	58.50	C_39_H_80_O_2_	580	–	4.8	–	–
39	3-Ethyl-5-(2-ethylbutyl)octadecane	61.06	C_26_H_54_	366	–	9.6	–	–
40	6,7-Epoxypregn-4-ene-9,11,18-triol-3,20-dione, 11,18-diacetate	63.71	C_25_H_32_O_8_	460	–	–	23.0	–
41	(22*E*)-Ergosta-5,22-dien-3-yl acetate	65.44	C_30_H_48_O_2_	440	–	6.3	–	–
**Total Identified**					**98.1**	**97.1**	**93.4**	**86.0**

MF = Molecular formula; MW = Molecular weight; RT = Retention time; C.C. = *C. comosum*; O.B. = *O. baccatus*; P.G. = *P. glutinosa*; A.M. = *A. monosperma*.

Altogether, 41 compounds were identified using GC chromatogram, retention time, peak area of each plant extract and comparing the mass spectra of each components with the library entries of mass spectra databases in NIST and Wiley libraries. Individually, 8, 12, 14 and 16 compounds were tentatively identified which accounts for 98.1%, 97.1%, 93.4% and 86.0% of total peak area of *C. comosum* stem, *O. baccatus* aerial parts, *P. glutinosa* stem and *A. monosperma* stem methanolic extracts, respectively. 3-(4-hydroxyphenyl) propionitrile **(1)**, 8,11-octadecadiynoic acid methyl ester **(2)**, 6,7-dimethoxycoumarin **(3)**, 1-(2-hydroxyphenyl) ethenone **(4)** (Fig. 5) were found to be the lead compounds of *C. comosum* stem, *O. baccatus* aerial parts *P. glutinosa* stem and *A. monosperma* stem, respectively (Fig. 4).

The prominent components present in the methanolic extracts of *C. comosum* stem were 3-(4-hydroxyphenyl) propionitrile (44.0%), 6-Pentyl-5,6-dihydro-2H-pyran-2-one (13.6%) and *n*-hexadecanoic acid methyl ester (7.5%). Whereas major components of methanolic extract of *O. baccatus* aerial parts were 8,11-octadecadiynoic acid methyl ester (33.9%), 3-ethyl-5-(2-ethylbutyl)octadecane (9.6%), 4-(2,6,6-trimethyl-1-cyclohexen-1-yl)-3-buten-2-ol (7.6%) and (*E*)-7-octadecene (7.6%) (Fig. 6). On the other hand, the major components of methanolic extracts of *P. glutinosa* stem were 6,7-dimethoxycoumarin (49.6%) and 6,7-epoxypregn-4-ene-9,11,18-triol-3,20-dione, 11,18-diacetate (23.0%) while in the methanolic extracts of *A. monosperma* stem main compounds were 1-(2-hydroxyphenyl) ethenone (10.3%), 2-[4-methyl-6-(2,6,6-trimethylcyclohex-1-enyl)hexa-1,3,5-trienyl]cyclohex-1-en-1-carboxaldehyde (9.1%), methyl 3-methylene-1,2,3,3a,4,4a,4b,5,6,10b-decahydrocyclopropa[3,4]cyclohepta[1,2-a]naphthalen-8-yl ether (8.2%) and 2-octadecyloxyethanol (7.5%) (Fig. 7).

## 4 Conclusion

In summary, the present *in vitro* cytotoxic screening of various Saudi medicinal plants has offered crucial preliminary information which potentially promotes the selection of plant species and their methanolic extracts with potential cytotoxic activities for future research. Among various plant species, *A. monosperma* stem, *O. baccatus* aerial parts, *P. glutinosa* stem, aerial parts of *C. arvensis* and *C. comosum* are good candidates for further phytochemical investigations of these plants. GG-MS analysis of most active plant extracts has resulted in the identification of 3-(4-hydroxyphenyl) propionitrile, 8,11-octadecadiynoic acid methyl ester, 6,7-dimethoxycoumarin, 1-(2-hydroxyphenyl) ethanone, as lead compounds from *C. comosum* stem, *O. baccatus* aerial parts, *P. glutinosa* stem and *A. monosperma* stem, respectively. However, further detail study on the chemical characterization of these phytomolecules and other compounds from these extracts using activity-guided fractionation and isolation approach for the identification of new anticancer compounds is required. Moreover, other plants such as methanolic extracts of *S. musilii* whole plants and *A. monosperma* leaves have also shown promising anticancer properties and could also be considered for future research for isolating cytotoxic compounds.

## Declaration of Competing Interest

The authors declare that they have no known competing financial interests or personal relationships that could have appeared to influence the work reported in this paper.
