# Supplementary material for: Screening of potential cytotoxic activities of some medicinal plants of Saudi Arabia
Source: Saudi J Biol Sci. 2021 Oct 25;29(3):1801–7. doi: 10.1016/j.sjbs.2021.10.045 (PMC8913393; doi:10.1016/j.sjbs.2021.10.045)
Supplement: Supplementary Data 1 [file mmc1.docx]

Screening of Potential Cytotoxic Activities of Some Medicinal Plants of Saudi Arabia

Merajuddin Khan*, Mujeeb Khan, Syed F. Adil and Hamad Z. Alkhathlan

*Department of Chemistry, College of Science, King Saud University, P.O.Box 2455 , Riyadh - 11451, Saudi Arabia.*

*Corresponding Author. *E-mail:* [mkhan3@ksu.edu.sa](mailto:mkhan3@ksu.edu.sa) (M. Khan).

*S1. Gas Chromatography (GC) and Gas Chromatography−Mass Spectrometry (GC-MS) Analysis of Plant Extracts*

GC–MS was performed on an Agilent single-quadrupole mass spectrometer with an inert mass selective detector (MSD-5975C detector, Agilent Technologies, USA) coupled directly to an Agilent 7890A gas chromatograph which was equipped with a split–splitless injector, a quickswap assembly, an Agilent model 7693 autosampler and a HP-5MS fused silica capillary column (5% phenyl 95% dimethylpolysiloxane, 30 m × 0.25 mm i.d., film thickness 0.25 μm, Agilent Technologies, USA). The column was operated using an injector temperature of 250°C and the following oven temperature profile: an isothermal hold at 50°C for 4 min, followed by a ramp of 4°C/min to 220°C, an isothermal hold for 2 min, a second ramp to 280°C at 20°C/min and finally an isothermal hold for 15 min.

Approximately 1.0 μl of each sample dissolved in methanol (HPLC grade) was injected using the split injection mode; the split flow ratio was 10:1. The helium carrier gas was flowed at 1 ml/min. The GC–TIC profiles and mass spectra were obtained using the ChemStation data analysis software, version E-02.00.493 (Agilent). All mass spectra were acquired in the EI mode (scan range of m/z 45–600 and ionization energy of 70 eV). The temperatures of the electronic-impact ion source and the MS quadrupole were 230°C and 150°C, respectively. The MSD transfer line was maintained at 280°C for the analysis. The GC analysis was performed on an Agilent GC-7890A dual-channel gas chromatograph (Agilent Technologies, USA) equipped with FID using nonpolar (HP-5MS) columns under the same conditions as described above. The detector temperature was maintained at 300°C for the analyses. The relative composition of the oil components was calculated on the basis of the GC–MS peak areas measured using the HP-5 MS column without using correction factor. Results are reported in Table S1-S4 according to their elution order on the HP-5MS column.

*S2. Identification of phytomolecules*

The identification of different components of most active methanolic extracts of four plants were done using their retention time, elution order and by matching the mass spectra with the library entries of mass spectra databases in NIST and Wiley libraries (WILEY 9th edition, NIST-08 MS library version 2.0 f).
